# Development and Translation of NanoBEO, a Nanotechnology-Based Delivery System of Bergamot Essential Oil Deprived of Furocumarins, in the Control of Agitation in Severe Dementia

**DOI:** 10.3390/pharmaceutics13030379

**Published:** 2021-03-12

**Authors:** Damiana Scuteri, Roberta Cassano, Sonia Trombino, Rossella Russo, Hirokazu Mizoguchi, Chizuko Watanabe, Kengo Hamamura, Soh Katsuyama, Takaaki Komatsu, Luigi Antonio Morrone, Laura Rombolà, Annagrazia Adornetto, Annarita S. Laganà, Maria Tiziana Corasaniti, Paolo Tonin, Shinobu Sakurada, Tsukasa Sakurada, Pierluigi Nicotera, Giacinto Bagetta

**Affiliations:** 1 Pharmacotechnology Documentation and Transfer Unit, Preclinical and Translational Pharmacology, Department of Pharmacy, Health and Nutritional Sciences, University of Calabria, 87036 Rende, Italy; damiana.scuteri@unical.it; 2 Regional Center for Serious Brain Injuries, S. Anna Institute, 88900 Crotone, Italy; patonin18@gmail.com; 3 Department of Pharmacy, Health and Nutritional Sciences, University of Calabria, 87036 Rende, Italy; roberta.cassano@unical.it (R.C.); sonia.trombino@unical.it (S.T.); annaritalag@virgilio.it (A.S.L.); 4 Preclinical and Translational Pharmacology, Department of Pharmacy, Health and Nutritional Sciences, University of Calabria, 87036 Rende, Italy; rossella.russo@unical.it (R.R.); luigi.morrone@unical.it (L.A.M.); laura.rombola@unical.it (L.R.); annagrazia.adornetto@unical.it (A.A.); 5 Department of Physiology and Anatomy, Tohoku Pharmaceutical University, 981-8558 Sendai, Japan; mizo@tohoku-mpu.ac.jp (H.M.); chizu@tohoku-mpu.ac.jp (C.W.); soukatsuyama@nichiyaku.ac.jp (S.K.); s-sakura@tohoku-mpu.ac.jp (S.S.); 6 Department of Pharmacology, Daiichi University of Pharmacy, 815-8511 Fukuoka, Japan; k-hamamura@daiichi-cps.ac.jp (K.H.); komatsu@daiichi-cps.ac.jp (T.K.); tsukasa@daiichi-cps.ac.jp (T.S.); 7 Department of Health Sciences, University “Magna Graecia” of Catanzaro, 88100 Catanzaro, Italy; mtcorasa@unicz.it; 8 German Center for Neurodegenerative Diseases (DZNE), 53127 Bonn, Germany; pierluigi.nicotera@dzne.de

**Keywords:** essential oil of bergamot, dementia, BPSD, agitation, pain, nanotechnology delivery system

## Abstract

Dementia is one of the most common causes of disability worldwide characterized by memory loss, cognitive impairment, and behavioral and psychological symptoms (BPSD), including agitation. Treatment of the latter consists of the off-label use of harmful atypical antipsychotics, though a significant reduction is afforded by pain control. The use of an essential oil endowed with analgesic properties and devoid of toxicity would represent an important option for the management of agitation in dementia. Therefore, the aim of this study was to engineer a nanotechnology delivery system based on solid lipid nanoparticles loaded with bergamot essential oil (BEO) and devised in the pharmaceutical form of an odorless cream (NanoBEO) to confirm its analgesic efficacy for further development and application to control agitation in dementia. BEO has proven strong antinociceptive and anti-allodynic properties and, in its bergapten-free form, it is completely devoid of phototoxicity. NanoBEO has been studied in vivo confirming the previously reported analgesic activity of BEO to which is now added its anti-itching properties. Due to the nanotechnology delivery system, the stability of titrated BEO components is guaranteed. Finally, the latter invention, currently under patent consideration, is smell-devoid allowing efficacy and safety to be established in double-blind clinical trials; until now the latter studies have been impeded in aromatherapy by the strong odor of essential oils. A clinical trial NCT04321889 has been designed to provide information about the efficacy and safety of NanoBEO on agitation and pain in patients suffering from severe dementia.

## 1. Introduction

The improvement of life expectancy over the 21st century has led to demographic aging with a steady increase of age-related neurodegenerative pathologies [1]. Alzheimer’s disease (AD) is the most widespread form of dementia estimated to affect some 75 million people by 2030 and to double by 2050 [2,3]. Chronic pain is often experienced by the elderly, ranging from 50% of community-dwelling older adults to 80% of care facilities residents, of whom 25% do not receive pain-relief [4,5,6]. The problem of pain can be even more burdensome when it coincides with severe dementia. In the real-world community setting, the access of these patients to pain treatment, particularly neuropathic, is very limited [7,8] and associated with the increased use of antipsychotics and antidepressants [9]. In fact, the inadequate pain treatment received by cognitively impaired patients is often due to their reduced or absent communication skills that prevent self-reporting: unrelieved pain is involved in the development of agitation [10], one of the most challenging behavioral and psychological symptoms of dementia (BPSD), also known as neuropsychiatric symptoms (NPS). This leads to the off-label use of antidepressants and atypical antipsychotics that double the risk of death for cardiocerebrovascular accidents in these patients [11]. Additionally, in the frame of the current novel severe acute respiratory syndrome coronavirus-2 (SARS-CoV-2) pandemic, it is the elderly who paid the highest price [12], and COVID-19 (COrona VIrus Disease 2019) and dementia, the pandemic of global aging [13], are overlapping. Pain assessment in severe dementia can be performed only through specific observational pain scales, but the risk of contagion often impedes these non-urgent practices [14]. This paints a picture of the worrying implications concerning the management of chronic pain in severe dementia and of its global social burden. A fundamental aspect to consider is that agitation can be remarkably minimized through adequate pain treatment and the regular review of therapy [15,16]. The epidemiology of pain in dementia highlights that its severity correlates significantly with dementia severity, NPS, and antipsychotic prescriptions [17]. Incidentally, pain and BPSD share some pathways in aged animals [18]. Thus, effective pain treatment is a priority and can avoid the unnecessary and harmful use of atypical antipsychotics, endowed with serious side effects, and often used off-label and without evidence of the benefits such as quetiapine. Only risperidone is licensed in Europe for demented patients suffering from resistant aggression and it can be used for a short period of up to six weeks, a maximum of twelve [19]. Aromatherapy with melissa and lavender has proven efficacy in the management of agitation in dementia [20,21], but clinical trials in aromatherapy are too few and small and most of them lack methodological consistency [22]. One of the points of weaknesses in the quality of the outcome assessment is due to the strong smell of essential oils that do not allow adequate allocation masking and double-blinding. Moreover, due to the tight link existing between pain and agitation, the essential oil to be used needs to be endowed with analgesic activity. Most preclinical studies on essential oils do not justify their clinical use, with the exception of the essential oil of bergamot (BEO, Citrus bergamia Risso et Poiteau) that has provided strong preclinical evidence of its antinociceptive and anti-allodynic effect [23]. BEO contains furocoumarins, e.g., bergapten, that can induce phototoxicity [24]; interestingly, BEO deprived of bergapten (BEO-bergapten free, BEO-BF) [25] keeps all its pharmacological properties and it is devoid of toxicity (European Medicine Agency (EMA), 13 September 2011 EMA/HMPC/56155/2011 Committee on Herbal Medicinal Products (HMPC)). Another issue with aromatherapy concerns the delivery system that does not allow the exposure of individuals to the known amount of phytocomplex, which hampers dose-effect relations in clinical settings for biological, therapeutic, or side effects and reproducibility. In addition, some volatile components of essential oils are relatively unstable to heat and light. Therefore, it is necessary to provide a delivery system of BEO able to overcome all these limitations. Here we present a nanotechnology BEO-BF delivery system, i.e., NanoBEO, obtained with the use of solid lipid nanoparticles (SLN) [26], in the pharmaceutical form of a cream for transdermic application, which is able to: deliver known amounts of the phytocomplex; to protect its ingredients from chemical instability; encapsulate the smell providing an odourless formulation. According to the present invention currently under patent consideration, it is now possible to effectively use aromatherapy with BEO-BF in complementary medicine for the therapy of acute and chronic pain and prevention or treatment of BPSD and any other stress, including itch-related behaviors and mood disorders in normal or demented people. The clinical trial NCT04321889 has been registered for the study of the effectiveness of NanoBEO on agitation and pain in severely demented patients. Therefore, the aim of this study was to engineer a nanotechnology delivery system based on solid lipid nanoparticles loaded with bergamot essential oil (BEO) and devised in the pharmaceutical form as an odorless cream (NanoBEO) to confirm its analgesic efficacy for further development and application to control agitation in dementia.

## 2. Materials and Methods

### 2.1. Reagents

All solvents were of analytical grade and purchased from Sigma-Aldrich (Sigma Chemical Co., St. Louis, MO, USA): tetrahydrofuran (THF), chloroform (CHCl3), *n*-hexane, ethyl acetate, dimethyl sulphoxide (DMSO), isooctane, 1-butanol, α-linolenic acid (PM = 278.43 g/mol), α-tocopherol (PM = 430.72 g/mol), biliary salt of the taurodeoxycholic acid, Tween 20, dicyclohexylcarbodiimide (DCC).

### 2.2. Essential Oil

BEO was kindly provided by “Capua Company1880 S.r.l.”, Campo Calabro, Reggio Calabria, Italy. The certificate of analysis indicates the following composition (Table 1):

The formulation is furocoumarin-free to avoid phototoxicity, according to the assessment report of the (EMA, HMPC) (13 September 2011 EMA/HMPC/56155/2011).

### 2.3. Preparation of the SLN with α-Tocopheryl Stearate (α-TFS): α-TFS-SLN

The α-TFS was synthesized as previously described [27]. Briefly, α-TF (1.0 g, 5.15 mmol), *p*-toluene sulfonic acid (0.07 g, 0.34 mmol), and stearyl alcohol (0.7 g, 5.4 mmol) was added to 10 mL dry toluene under stirring and N_2_ at room temperature. The solution was stirred under N_2_ for 12 h at 110 °C. After cooling at room temperature, the solvent was evaporated under reduced pressure, the residue treated with 15mL water, and the aqueous phase was extracted with chloroform (4 × 15mL). The combined organic phases were collected and dried using sodium sulfate (Na_2_SO_4_), a common inorganic drying agent that acquires water hydration when exposed to moist air or a wet solution. Afterward, the solvent was removed by rotary evaporation to give a yellow-colored α-TFS and it was purified by Merck silica gel (60–230 mesh) column chromatography, using ethylacetate/*n*-hexane85/15 (*v*/*v*) as the eluent. The solvent of eluated ester was evaporated under reduced pressure. The α-TFS was identified by TLC, UV, and 1H-NMR analyses [27,28,29]. The α-TFS-SLN were prepared by a microemulsion technique at a moderate temperature [30]. The α-TFS (142 mg, 0,201 mmol) was mixed with BEO-BF by heating at a temperature lower than temperatures that degrade BEO, preferably in the range of 60–65 °C. Then, a warm water (0.21 mg, 6% of α-TFS 0.0012 mmol) solution of sodium taurodeoxycholate (25.33 mg, 30% α-TFS, 0.061 mmol), butanol (0.3 mg, 2% of α-TFS, 0.0041 mmol), and Tween 20 (75 mg, 30% of α-TFS, 0.061 mmol) was added to obtain an optically transparent microemulsion. Sodium taurodeoxycholate and Tween 20 acted as emulsifiers and butanol as co-emulsifier. The warm microemulsion was immediately dispersed in cold water (2 °C) under high-speed homogenization (Model SL2, Silverson, Chesham Bucks, England) at 8000 rpm for 30 min (240,000 g in 30 min). The volume ratio of warm microemulsion to cold water was 1:20. The α-TFS-SLN dispersions were washed twice using an Amicon TCF2A ultrafiltration system (Amicon Grace, Beverley, MA, USA; membrane Amicon Diaflo YM 100).

### 2.4. Size Distribution Analysis

The size of the α-TFS-SLN was determined by dynamic light scattering using a 90 Plus Particle Size Analyzer (Brookhaven Instruments Corporation, Holtsville, NY, USA) at 25 °C by measuring the autocorrelation function at a 90° scattering angle. Cells were filled with 100 µL of the sample solution and diluted to 4mL with filtered (0.22 µm) water. The polydispersity index indicating the measure of the distribution of the nanoparticle population was also determined [31].

### 2.5. Morphology of SLN

**Figure 1:**
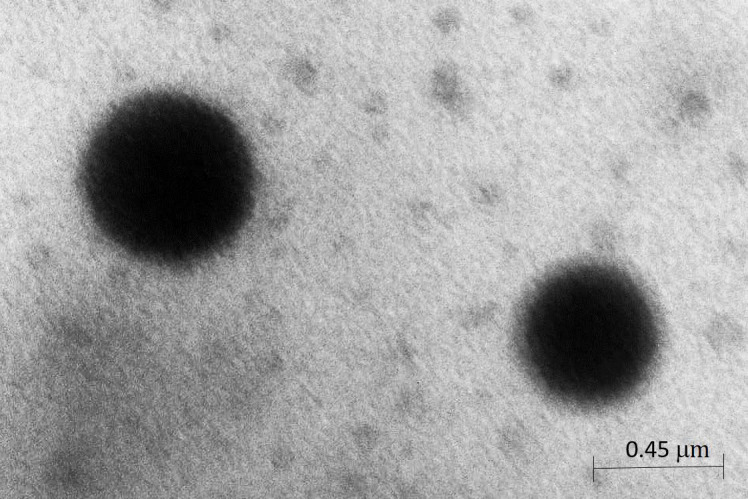
Photomicrograph of solid lipid nanoparticles (SNL) with α-tocopheryl stearate (α-TFS-SLN) visualized by transmission electronic microscopy (TEM).

The morphology of the hydrated SLN dispersions was examined using transmission electron microscopy (TEM). In particular, a drop of SLN dispersion was applied to a carbon-coated copper grid and left for 1 min to allow some of the particles to adhere to the carbon substrate. The excess of dispersion was removed by adsorbing the drop with a piece of filter paper. A drop of 1% phosphotungstic acid solution was applied and, again, the excess of the solution was removed by adsorbing the liquid with the tip of a filter paper and the sample was air-dried. The sample was observed under a ZEISS EM 900 electron microscope (Oberkochen, Germany) at an accelerating voltage of 80 kV (Figure 1).

### 2.6. Percentage of BEO-BF Incorporated into α-TFS-SLN

To evaluate BEO-BF content, the α-TFS-SLN formulation (1mL) was diluted to 10 mL with methanol and analyzed by spectrophotometric detection at wavelengths of 281 nm for linalool, 208 nm for linalyl acetate, and 247 nm for limonene.

#### Preparation of a Nanotechnology Delivery System (NDS) Based on α-TFS-SLN Containing BEO-BF

The composition of 44.227 g of cream based on α-TFS-SLN containing BEO-BF is reported below:

- 37.604 g of a purified water suspension of α-TFS-SLN containing BEO-BF;
- 4.42 g of sweet almond oil;
- 0.885 g of polyacrylamide;
- 0.442 g of isoparaffin C13-14;
- 0.111 g of 7-laurate;
- 0.774 g of purified water Ph.Eur.;
- 0.028 g of methylparaben;
- 0.009 g of propylparaben.

### 2.7. Animals

Male ddY (SD) mice (Shizuoka Laboratory Center, Japan; Japan SLC, Hamamatsu, Japan; Kyudo Industries, Kumamoto, Japan) weighing 22–26 g at the time of testing were used. The mice were individually housed in a colony maintained in a controlled environment (12 h light/dark cycle, room temperature 23 °C, 50–60% relative humidity). The animals had free access to food pellets and water. All behavioral experiments took place during the light period between 10:00 and 17:00 h in a quiet room. The animals were tested in a randomized order. All experiments were performed following the approval of the Ethics Committee for Animal Experiments of the Daiichi University of Pharmacy and the Tohoku Pharmaceutical University (Examination number of Daiichi University of Pharmacy: H29-005, approval number: 17003; 30 June 2017) and in accordance with the National Institutes of Health Guides for the Care and Use of Laboratory Animals [32]. The experimental design and statistical power analysis were established to minimize the number of animals used while still generating reliable results, to meet with the scope of the 3R approach to refine, reduce, and, at least in part, replace. According to the statistical power calculation based on similar studies in the literature, *n* = 5 animals per group subjected to analgesic treatment was sufficient to obtain a 30% reduction of nociceptive reaction.

### 2.8. Experimental Models of Pain and Itch

#### 2.8.1. Capsaicin Test

Antinociception was assessed using the capsaicin test as previously described [33,34]. To reduce variability, each mouse was acclimatized to an acrylic observation chamber (22.0 × 15.0 × 12.5 cm) for approximately 1 h before the injection of capsaicin. The mouse was injected with 20 μL of a solution of capsaicin (0.8 μg/paw) beneath the skin of the plantar surface of the right hind paw using a 50 μL Hamilton microsyringe with a 26-gauge needle as quickly as possible, with only minimal animal restraint. Following capsaicin injection, the animals were immediately placed in the test box for a 5-min observation period. Licking/biting behavior induced by the intraplantar injection of capsaicin was observed as an indicator of nociceptive response. NanoBEO (1 mg/mouse) and control (1 mg/mouse) was applied around the plantar surface of the right hind paw using a cotton swab 30 min prior to the capsaicin injection. The accumulated response time in seconds spent in licking/biting the capsaicin-injected hind paw was measured for a period of 5 min immediately after the subcutaneous (s.c.) injection of capsaicin.

#### 2.8.2. Formalin Test

Mice were placed into a transparent cage (22.0 cm × 15.0 cm × 12.5 cm high) which also served as an observation chamber and were allowed to adapt to their environment for 1 h before testing. After this period, a plantar subcutaneous injection of 20 μL formalin (0.5% in saline) using a microsyringe with a 26-gauge needle was performed [35]. The low concentration of 0.5% formalin produced only the phasic (acute) paw-licking response, lasting the first 5 min after the formalin injection. NanoBEO (1 mg/mouse) and control (1 mg/mouse) was applied around the plantar surface of the right hind paw using a cotton swab 30 min prior to the formalin injection. Each mouse was immediately returned to the observation chamber after formalin injection. The accumulated response time in seconds spent in licking and biting of the injected hind paw was defined as a nociceptive response and the total time (s) of the response was measured with a hand-held stop-watch for a period of 5 min immediately after the subcutaneous (s.c.) injection of formalin.

#### 2.8.3. Partial Sciatic Nerve Ligation (PSNL)

Mice were anesthetized by isoflurane (2.0%, FUJIFILM Wako Pure Chemical Corporation, Osaka, Japan). NanoBEO and control were administered on postoperative day 7. The sciatic nerve of the right hindlimb was exposed at the high thigh level through a small incision and the distal one-third to one-half of the dorsal portion of the sciatic nerve was tied with non-absorbable silk thread [36]. The wound was closed with silk thread suture and covered with antibiotic powder. Immediately following surgery, the animals were kept in a soft bag cage with some food inside so that they could feed themselves without having difficulty standing. The wound healed within 1 to 2 days, and the mice with ligated nerves did not present signs of foot clonus or autotomy but behaved normally. The presence of mechanical allodynia (sensitization) was determined by the von Frey test. Animals were allowed to habituate to the testing environment and Plexiglass observation chamber (9.0 × 9.0 × 14.0 cm, length × width × height, Ugo Basile, Gemonio, Italy) with a wire mesh floor for approximately 1 h prior to testing. Calibrated von Frey filaments (pressure stimulus 0.40 g, Natsume Seisakusho Co., Ltd., Tokyo, Japan) were then applied to the right plantar surface of the hind paw of the mice. The paw withdrawal threshold was evaluated using the up-down method. NanoBEO (1 mg/mouse) and control (1 mg/mouse) was applied around the plantar surface of the right hind paw using a cotton swab 30 min prior to the von Frey test. All the testing was performed by a blind observer.

#### 2.8.4. Itch Test

The effect of NanoBEO was also studied on the scratching behavior induced by 4-methyl-histamine administration [37]. Scratching belongs to behavioral disturbances shown by patients affected by dementia. Mice have been pretreated with NanoBEO BEO-BF cream (1 mg/mouse) or control cream (1 mg/mouse) for transdermal application 30 min prior or immediately before intradermal administration of 4 methyl-histamine (200 µg/µL) and the scratching behavior was filmed for 30 min and measured offline by an independent observer. The 4-methyl-histamine is a pharmacological tool used to induce itching behavior in mice. The latency time was 10 min. The occurrence of scratching behavior in the mice subjected only to 4-methyl-histamine administration was observed. This behavior was more intense in the first minute of observation, but it occurred also from 2 min and 40 s and was completely prevented by the topical application of NanoBEO.

### 2.9. Statistical Analysis

Nociceptive behavior for each treatment group was expressed as mean ±S.E.M. Statistical differences between the groups was established by using the Student’s *t*-test (GraphPad Prism; GraphPad Software 6, Inc., San Diego, CA, USA). Values of *p* < 0.05 were considered statistically significant.

## 3. Results

### 3.1. Size Distribution Analysis and Morphology of SLN

The morphology of the hydrated SLN demonstrated that it was successfully prepared by the microemulsion technique. They showed a diameter equal to 450 nm and a polydispersity index of 0.30. Photomicrographs of α-TFS-SLN visualized by TEM are shown in Figure 1.

### 3.2. Percentage of BEO-BF Incorporated into α-TFS-SLN

To evaluate BEO-BF content, the α-TFS-SLN formulation (1 mL) was diluted to 10 mL with methanol and analyzed by spectrophotometric detection at wavelengths of 281 nm for linalool, 208 nm for linalyl acetate, and 247 nm for limonene. The results indicated the presence of 27% of linalool, 31% of linalyl acetate, and 36% of limonene. After two and six months of light exposure, these percentages declined by 10% and 18%, respectively, with no further degradation at twelve months, demonstrating that α-TFS-SLNs effectively slowed down the degradation of BEO-BF.

### 3.3. Antinociceptive Effect of NanoBEO

**Figure 2:**
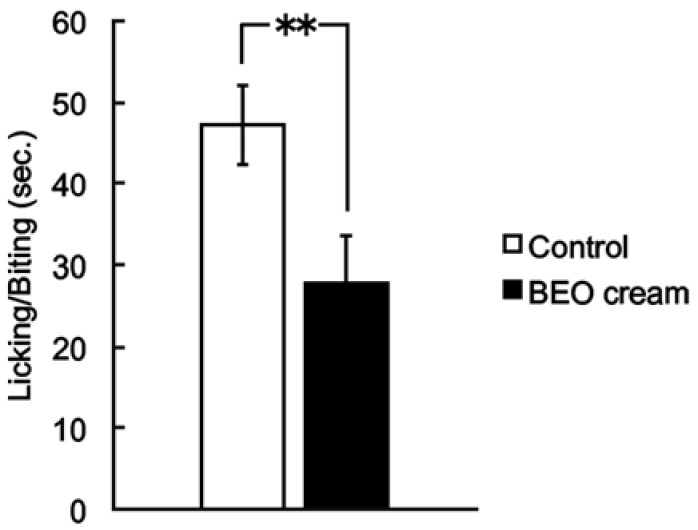
Antinociceptive effect of topical application of NanoBEO (1 mg/mouse) on capsaicin-induced licking/biting behavior. The duration of licking/biting induced by capsaicin has been determined using the 5-min period starting immediately after the injection of capsaicin. Empty cream (1 mg/mouse) has been used as a control and this has failed to affect the capsaicin-induced nociceptive response. Values are expressed as mean ± SEM (*n* = 8). Values of *p* < 0.05 have been considered statistically significant. Improvement over control of 59.26%, ** *p* < 0.01.

**Figure 3:**
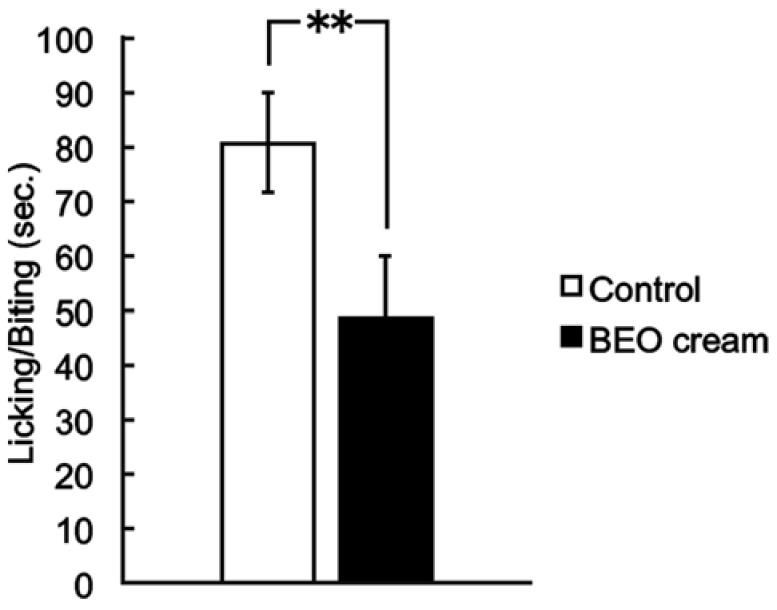
Antinociceptive effect of the topical application of NanoBEO (1 mg/mouse) on the 0.5% formalin test. The nociceptive behavior induced by formalin has been scored as the amount of time spent licking and biting the injected paw. Empty cream (1 mg/mouse) has been used as a control and this has failed to affect the formalin-induced nociceptive response. Values are expressed as mean ± SEM (*n* = 8). Values of *p* < 0.05 have been considered statistically significant. Improvement over control of 59.78%, ** *p* < 0.01.

**Figure 4:**
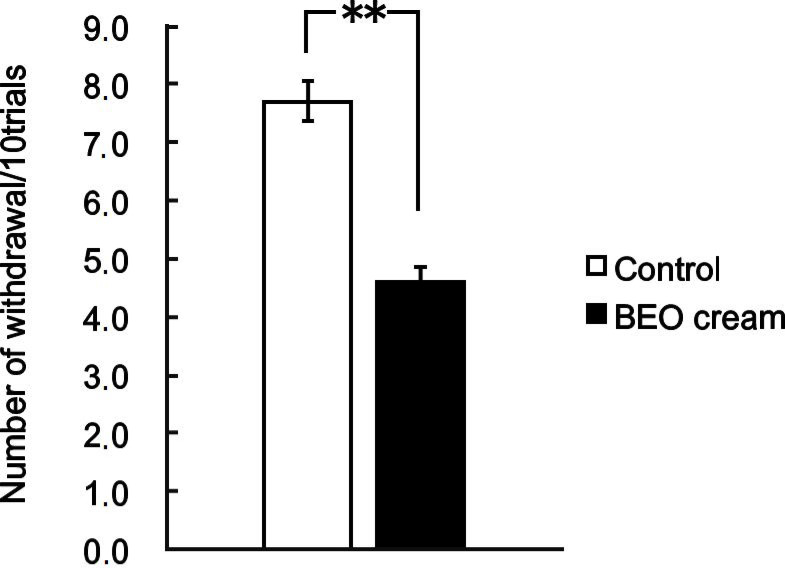
Antiallodynic effect of topical application of NanoBEO (1 mg/mouse) on partial sciatic nerve ligation (PSNL). Empty cream (1 mg/mouse) has been used as a control and this has failed to affect PSNL-induced mechanical allodynia. Each value represents mean ± SEM (*n* = 10). Values of *p* < 0.05 have been considered statistically significant. Improvement over control of 58.76%, ** *p* < 0.01.

**Figure 5:**
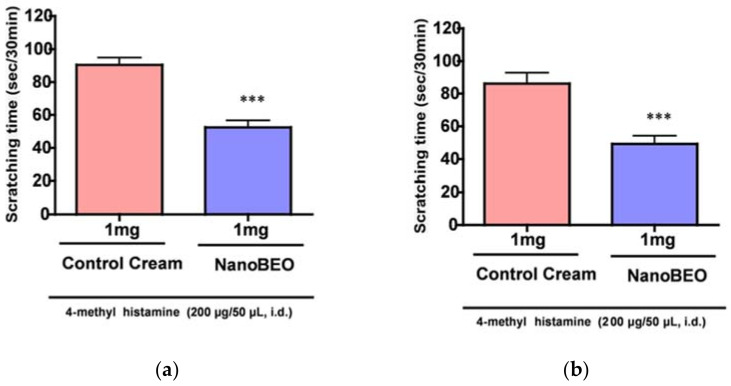
Anti-scratching effect of intradermal (i.d.) administration of NanoBEO on 4-methyl-histamine-induced behavior. The NanoBEO (1 mg/mouse) was used as pretreatment (30 min) (**a**) or immediately after (**b**) and has resulted as effective on the scratching behavior under both circumstances. The scratching time has been scored as seconds per 30 min. Empty cream (1 mg/mouse) has been used as a control and this has failed to affect itching. Values are expressed as mean ± SEM (*n* = 8). Values of *p* < 0.05 have been considered statistically significant. (**a**) Improvement over control of 53.13%, *** *p* < 0.001; (**b**) improvement over control of 56.67%, *** *p* < 0.001.

As demonstrated for BEO, NanoBEO has proven analgesic efficacy both in the inflammatory pain models of the capsaicin (59.26% improvement over control, ** *p* < 0.01; Figure 2) and the formalin test (59.78% improvement over control, ** *p* < 0.01; Figure 3) and in the neuropathic pain model of the PSNL (58.76% improvement over control, ** *p* < 0.01; Figure 4). In comparison with the empty NDS (control), NanoBEO was also effective in preventing 4-methyl-histamine-induced scratching behavior (Figure 5a,b). The effect of NanoBEO on itching is reported in a short movie within the Supplementary Materials.

## 4. Discussion

NanoBEO, consisting of an odorless NDS of α-TFS-SLN loaded with BEO-BF, has been tested on acute and neuropathic pain models confirming the strong antinociceptive and anti-allodynic efficacy previously reported for BEO. In fact, BEO has demonstrated analgesic properties in acute pain models, e.g., the capsaicin test [33,34] and early phase of the 0.5% formalin test [38], but also in neuropathic pain models, like the partial sciatic nerve ligation [36,39,40] and the spinal nerve ligation [25]. It enhances morphine-induced analgesia and its dose-dependent effect is antagonized by naloxone hydrochloride and methiodide (not crossing the blood-brain barrier), suggesting the involvement of peripheral opioid mechanisms. Quite importantly, BEO is able to modulate the synaptic level of glutamate [41], involved in pain transmission and modulation through the pain descending pathway due to metabotropic glutamate receptors [3]. Its analgesic efficacy occurs also via the inhalation route [42], but it has been demonstrated not to depend on inhalation [40]. This is fundamental since patients suffering from dementia frequently present dysfunctional olfaction [43] and neurofibrillary tangles in the entorhinal cortex [44]. Furthermore, BEO is characterized by anxiolytic effects not superimposable to those of diazepam [45] and insensitive to flumazenil [16], hence devoid of sedation and involving serotonergic transmission [46]. The latter pharmacologic properties are very important for the use in dementia since benzodiazepines can worsen cognitive function bearing an anti-cholinergic burden [47]. Moreover, it is effective also on scratching behavior, one of the behavioral disturbances displayed by patients suffering from dementia. The results obtained testing the nanotechnology BEO delivery system showed stable and reproducible aromatherapy effects. In particular, after two and six months of light exposure, the content in the active ingredients declined by 10% and 18%, respectively with no further degradation at twelve months. In fact, these α-tocopheryl stearate-based SLN contain ferulic acid as an anti-oxidant agent, differently from those previously described (US5250236) devoid of anti-oxidants. Therefore, the prolonged physicochemical stability (up to 12 months) of the phytocomplex titrated in its main components (linalool, linalyl acetate, and limonene) is a remarkable advantage allowing reproducible antinociceptive and anti-itch responses to be measured. This latter property is of primary importance because components of the phytocomplex are implicated in the analgesic action of BEO. In fact, s.c. administration of linalool was demonstrated to reduce licking/biting response to formalin [38] and to capsaicin [34] by means of an opioid receptor-dependent mechanism. Linalool attenuates paclitaxel-induced mechanical allodynia and hyperalgesia [48]. The anti-allodynic effect was also reported in the spinal nerve ligation model [49]. Blockade of the activation of spinal extracellular signal-regulated protein kinase (ERK) occurring during neuropathic pain induction underscores the anti-allodynic effect of linalool [36]. Incidentally, BEO enhances basal and induced autophagy, a process implicated in dementia [50,51] and pain [52] and this has been attributed to limonene [53]. Moreover, β-caryophyllene is a common component of essential oils, among which is BEO, and it is endowed with an antinociceptive effect demonstrated in the capsaicin test, mediated by activation of CB2 receptors [54]. This NDS has the features to guarantee a significant reduction of the total loss of transepidermal water and a large surface area of the SLN, which allows the prolonged exposure and contact of the loaded BEO with the skin, resulting in an efficient release. Added to this is the possibility to perform double-blind clinical trials, impossible so far because of the strong smell of essential oils used in aromatherapy. The diagnosis and treatment of pain in cognitively impaired patients is a neglected area, from chronic pain in dementia and migraine in aged patients often affected by concurrent cognitive impairment to post-stroke pain. Demented patients receive limited treatment for chronic pain, particularly neuropathic [9]. Novel preventative treatments for migraine like the anti-calcitonin gene-related peptide (CGRP) monoclonal antibody eptinezumab that can provide rapid and longer-lasting action [55] are not studied in these patients. Patients suffering from post-stroke pain are often subjected to cognitive deficits and clinical trials studying the use of the strongest painkillers, i.e., opioids, in this population are very few and not adequately powered and designed [56]. This is often due to the lack of use of observational tools for non-communicative patients and this problem is worsened by the current coronavirus pandemic. Therefore, the registered clinical trial NCT04321889 BRAINAID (Bergamot Rehabilitation AgaINst Agitation In Dementia) has been designed to assess the effectiveness of NanoBEO on agitation and pain in severely demented patients to offer a safe tool able to provide relief to this fragile population. It will enroll 134 patients aged ≥65 years with severe dementia (Mini-Mental State Examination [MMSE] <12) who will be randomized to NanoBEO or placebo cream in a 1:1 allocation ratio. The primary endpoint is the assessment of agitation through the Cohen-Mansfield Agitation Inventory (CMAI) over four weeks. The secondary endpoints will be the 4-week follow-up of the effect on agitation and the effect on pain measured with the Mobilization–Observation–Behaviour–Intensity–Dementia (MOBID)-2. This double-blind clinical trial will be the first to assess the efficacy and safety of an engineered essential oil with proven strong analgesic properties in acute and neuropathic pain on agitation. The latter trial can provide the rationale for the safer treatment of BPSD and pain and it can confirm the analgesic properties of BEO in clinic.

## 5. Conclusions

In conclusion, our data lend support to the use of the engineered nanotechnology delivery system based on SLN loaded with BEO-BF and devised in the pharmaceutical form of an odorless cream (NanoBEO) in the control of agitation in dementia. In fact, NanoBEO maintains the analgesic properties of the phytocomplex and it is smell-devoid, now allowing efficacy and safety to be established in double-blind clinical trials in demented patients.

## 6. Patents

The present invention is currently under patent consideration at national (deposit N. 102019000013353) and international (deposit N. WO2021019588) level.

## Figures and Tables

**Table 1:** Composition of bergamot essential oil (BEO) according to the certificate of analysis.

Chemical Substance	Interval Ranges (%)
α-Pinene	0.7–2.0
Sabinene	0.5–2.0
β-Pinene	5.0–10.0
Limonene	30.0–50.0
γ-Terpinene	6.0–18.5
Linalool	6.0–15.0
Linalyl acetate	23.0–35.0
Geranial	<0.5
Geranyl acetate	0.1–0.7
Cariophyllene	0.2–0.5

## Data Availability

The data presented in this study are available within the article or supplementary material.

## Supplementary Materials

The following are available online at https://www.mdpi.com/1999-4923/13/3/379/s1, Video S1: Supplementary material short movie.
